# Antimicrobial Resistance and Molecular Characterization of *Staphylococcus aureus* Causing Childhood Pneumonia in Shanghai

**DOI:** 10.3389/fmicb.2017.00455

**Published:** 2017-03-21

**Authors:** Zhen Song, Fei-Fei Gu, Xiao-Kui Guo, Yu-Xing Ni, Ping He, Li-Zhong Han

**Affiliations:** ^1^Faculty of Laboratory Medicine, Ruijin Hospital, Shanghai Jiao Tong University School of MedicineShanghai, China; ^2^Department of Clinical Microbiology, Ruijin Hospital, Shanghai Jiao Tong University School of MedicineShanghai, China; ^3^Department of Immunology and Microbiology, Institutes of Medical Sciences, Shanghai Jiao Tong University School of MedicineShanghai, China

**Keywords:** MRSA, *Staphylococcus aureus*, antimicrobial resistance, molecular characterization, childhood pneumonia

## Abstract

*Staphylococcus aureus* or methicillin-resistant *Staphylococcus aureus* (MRSA) is a major pathogen causing pneumonia among children. To estimate the prevalence and molecular properties of *S. aureus* in children pneumonia in Shanghai, China, 107 hospitalized children with *S. aureus* pneumonia from two children's hospitals from January 2014 through June 2015 were studied. *S. aureus* isolates from the respiratory specimens were characterized by antimicrobial susceptibility, *agr* typing, toxin genes, multilocus sequence typing (MLST), *spa*, and SCCmec typing. Fifty-eight (54.2%, 58/107) were MSSA (methicillin-susceptible *Staphylococcus aureus*) and 49 (45.8%, 49/107) were MRSA. No isolates were found resistant to teicoplanin, sulfamethoxazole/trimethoprim, rifampicin, quinupristin/dalfopristin, linezolid, or vancomycin. However, these isolates showed high resistant rates to erythromycin, fosfomycin-trometamol and clindamycin. The *agrI* (87/107, 81.3%) was the most common *agr* allele, followed by *agrIII*(10/107, 9.3%), *agrII*(9/107, 8.4%), and *agrIV*(1/107, 0.9%). Six *pvl*-positive isolates (3 MRSA and 3 MSSA) and 7 isolates of livestock associated clone ST398 (4 MRSA, 3 MSSA) were identified. CC59 was found in 35 isolates (33 MRSA and 2 MSSA), constituting majority of MRSA (33/49, 67.35%). The dominant CC were CC59 (32.7%), CC188 (13.1%), CC7 (12.1%) and CC398 (9.3%) while t172 (16.8%), t189 (12.1%), t437 (9.3%), and t091 (9.3%) were the most common *spa* types. In conclusion, more particular concern should appeal to ST59-SCCmecIV-t172/t437 as it is the most common epidemic clone causing pneumonia among children in Shanghai.

## Introduction

Pneumonia causes high mortality among children under 5 years old. According to data from the World Health Organization, there are more children died from pneumonia than AIDS, malaria and measles combined (Adegbola, 2012). In industrialized countries, 1–10% of community-acquired and 20–50% of nosocomial bacterial pneumonia in children were caused by *S. aureus* (Bradley, 2005; Chisti et al., 2009). First reported in 1961, soon after the clinical introduction of methicillin, MRSA (methicillin-resistant *Staphylococcus aureus*) has become major nosocomial pathogen worldwide, and was found in the community (community-associated MRSA, CA-MRSA) in the late 1980s. Four pediatric necrotizing pneumonia caused by CA-MRSA in 1999 raised a global public health concern (Centers for Disease Control and Prevention, 1999). Now it has been estimated that up to 40% of patients with CA-MRSA had severe or fatal pneumonia (Wallin et al., 2008; Carcillo et al., 2009).

Several CA-MRSA clones have been reported with different regional prevalence, while ST30 clones are found worldwide (Aires De Sousa et al., 2003; Hsu et al., 2006). ST8 (USA300) and ST1 (USA400) are mostly reported in the United States and Canada (Mulvey et al., 2005; Tenover et al., 2006), ST80 is the prevalent clone in Europe (Holmes et al., 2005; Wannet et al., 2005; Fossum and Bukholm, 2006), and ST59 spreads mostly in the Asian-Pacific area (Coombs et al., 2004; Ho et al., 2007; Huang and Chen, 2011).

In recent years, some researchers reported MRSA infections in Chinese children. ST239 and ST59 were common clones in hospital- associated infections caused by MRSA (Ning et al., 2015). However, rare data of prevalence, antimicrobial susceptibility and molecular epidemiology of MRSA causing childhood pneumonia were available in Shanghai. We conducted a study on the resistance phenotype and sequence types (STs) of *S. aureus* isolates from two pediatric hospitals in Shanghai.

## Materials and methods

### Study design

This study was conducted in two tertiary teaching children's hospitals in Shanghai, offering comprehensive pediatric in-patient and out-patient services for approximately 21,000 inpatients and 1.25 million outpatients from Shanghai metropolitan area and eastern provinces annually. The two hospitals are located Pudong and Puxi districts respectively, being 14.1 kilometers apart from each other. One hundred and seven pediatric inpatients with pneumonia caused by *S. aureus* from January 2014 to June 2015 were enrolled in the study. Diagnosis of pneumonia are based on clinical presentations (acute clinical deterioration, pulse oximetry, increased respiratory support requirement), radiological findings (presence of new or changing infiltrate on chest radiography) and laboratory tests (elevated C-reactive protein or abnormal white blood cell count).

One hundred and seven *S. aureus* isolates from 107 children with pneumonia were collected, which were identified by combination of phenotypic tests as previously described (Chen et al., 2012).

This study was approved by Ruijin Hospital Ethics Committee (Shanghai Jiao Tong University School of Medicine), and the Review Board exempted the need for informed consent because this retrospective study mainly focused on bacteria and did no interventions to patients.

### Clinical data

A total of 107 *S. aureus* isolates were identified. The sample types included throat swabs (1/107, 0.9%), sputum (104/107, 97.2%) and bronchoalveolar lavage (2/107, 1.9%). *S. aureus* isolated from sputum represented lower respiratory tract there are 25 PMN and less than 10 epithelial cells per low power field. Seventy-one males and 36 females were enrolled and their median age was 51 days (range: 3–1,825 days).

### Antimicrobial susceptibility tests

The antimicrobial susceptibility tests were conducted by disk diffusion method according to the guidelines of CLSI M100-S25 (Patel et al., 2015). The antibiotics included penicillin (10 units), cefoxitin (30 μg), gentamicin (10 μg), kanamycin (30 μg), tobramycin (10 μg), fosfomycin-trometamol (200 μg), erythromycin (15 μg), tetracycline (30 μg), teicoplanin (30 μg), minocycline (30 μg), ciprofloxacin (5 μg), clindamycin (2 μg), sulfamethoxazole-trimethoprim (25 μg), chloramphenicol (30 μg), rifampicin (5 μg), quinupristin-dalfopristin (15 μg) and linezolid (30 μg). The penicillin disk diffusion zone edge test was used for β-lactamase detection, and inducible clindamycin resistance was examined by the *D*-test. The minimum inhibitory concentration (MIC) of vancomycin was detected by the agar dilution method. *S. aureus* ATCC25923 and ATCC29213 were used for quality control.

### Molecular typing

DNA was extracted by the simplified alkaline-lysis method (Chen et al., 2012). All *S. aureus* isolates were performed with *spa* typing, accessory gene regulator (*agr*) typing and multilocus sequence typing (MLST) (Chen et al., 2013). MRSA strains were confirmed by presence of the *mec*A gene, and SCC*mec* types of MRSA were determined as previously described (Ito et al., 2014).

### Detection of toxin genes

The Toxin gene profiles were performed by PCR, and candidate genes includes *lukS/F-PV* (encoding Panton-Valentine leukocidin); *tst* (encoding toxic shock syndrome toxin 1); *eta* and *etb* (encoding exfoliative toxin A and B); *sea*-*see* and *seg*-*sej* (encoding staphylococcal enterotoxins SEA-SEE and SEG-SEJ) (Jarraud et al., 2002), and *sasX* (encoding a mobile genetic element) which also acts as a virulence determinant and plays a key role in MRSA colonization and pathogenesis (Li et al., 2012).

### Statistical analysis

SPSS 22.0 (SPSS Inc., Chicago IL, USA) was used for Data analysis, including the chi-square or Fisher's exact test. A two-sided *p* < 0.05 was considered to be statistically significant.

## Results

### Antimicrobial susceptibility tests

Among 107 *S. aureus* isolates, 49 (45.8%) were MRSA, and 58 (54.2%) were methicillin-susceptible *S. aureus* (MSSA) according to CLSI M100-S25 guidelines. All isolates were susceptible to teicoplanin, sulfamethoxazole-trimethoprim, rifampicin, quinupristin-dalfopristin, linezolid and vancomycin. Only one MRSA isolate was intermediate to minocycline. Three ciprofloxacin-resistant (2.8%) and 14 ciprofloxacin-intermediate (13.1%) isolates were found. The ciprofloxacin-resistant isolates belonged to ST764, ST88, and ST2315, while all ciprofloxacin-intermediate isolates were ST59-SCCmecIV-t172. Seven (6.5%) penicillin-susceptible isolates were β-lactamase positive. A total of 20 isolates (10 MRSA and 10 MSSA) were inducible resistance to clindamycin as determined by *D*-test. MSSA isolates had significantly higher susceptibility than MRSA isolates in the following antibiotics: penicillin, tobramycin, erythromycin, clindamycin and ciprofloxacin (Table 1).

**Table 1:** **Antimicrobial susceptibilities of ***S. aureus*** isolated from children with pneumonia**.

**Antimicrobial**	**Susceptibilities rate (%)**			***P*-value**
	**Overall (*n* = 107)**	**MSSA (*n* = 58)**	**MRSA (*n* = 49)**	
Penicillin	–	7 (12.1)	–	–
Gentamicin	104 (97.2)	56 (96.6)	48 (98.0)	1.000
Kanamycin	80 (74.8)	46 (79.3)	34 (69.4)	0.239
Tobramycin	91 (85.0)	45 (77.6)	46 (93.9)	0.019
Fosfomycin-trometamol	42 (39.3)	24 (41.4)	18 (36.7)	0.624
Erythromycin	52 (48.6)	43 (74.1)	9 (18.4)	0.000
Tetracycline	92 (86.0)	50 (86.2)	42 (85.7)	0.942
Teicoplanin	107 (100)	58 (100)	49 (100)	–
Minocycline	106 (99.1)	58 (100)	48 (98.0)	0.458
Ciprofloxacin	90 (84.1)	55 (94.8)	35 (71.4)	0.001
Clindamycin^a^	74 (69.2)	54 (93.1)	20 (40.8)	0.000
Sulfamethoxazole-trimethoprim	107 (100)	58 (100)	49 (100)	–
Chloramphenicol	103 (96.3)	57 (98.3)	46 (93.9)	0.494
Rifampicin	107 (100)	58 (100)	49 (100)	–
Quinupristin-dalfopristin	107 (100)	58 (100)	49 (100)	–
Linezolid	107 (100)	58 (100)	49 (100)	–
Vancomycin^b^	107 (100)	58 (100)	49 (100)	–

a *20 isolates (10 MRSA and 10 MSSA) were D-test positive, indicating inducible clindamycin resistance*.

b *MIC range, 0.25~0.5 μg/ml; 85 isolates (48 MSSA and 37 MRSA) MIC = 0.25 μg/ml; 22 isolates (10 MSSA and 12 MRSA) MIC = 0.5 μg/ml*.

### Virulence factors

The *seb* (42/107, 39.3%) was the most frequent toxin gene, followed by *sea* (28/107, 26.2%). No *etb, see, sasX* were found. *sec, sed, she*, and *sej* were only found in MSSA isolates. There was a significant difference between MRSA and MSSA isolates in prevalence among the toxin genes of *sea, seb, seg*, and *sei* (Table 2). Six *S. aureus* isolates carried *lukS/F-PV* gene, and three of which were MRSA.

**Table 2:** **Prevalence of toxin genes among ***S. aureus*** from children with pneumonia**.

**Toxin gene**	**No. of positive isolates (% of 107)**	**No. distributing in**		***P***
		**MSSA (*n* = 58) *n* (%)**	**MRSA (*n* = 49) *n* (%)**	
*lukS/F-PV*	6 (5.6)	3 (5.2)	3 (6.1)	1.000
*tst*	4 (3.7)	3 (5.2)	1 (2.0)	0.734
*eta*	3 (2.8)	2 (3.4)	1 (2.0)	1.000
*etb*	0	0	0	–
*sea*	28 (26.2)	9 (15.5)	19 (38.8)	0.006
*seb*	42 (39.3)	9 (15.5)	33 (67.3)	0.000
*sec*	4 (3.7)	4 (6.9)	0	0.173
*sed*	3 (2.8)	3 (5.2)	0	0.304
*see*	0	0	0	–
*seg*	11 (10.3)	10 (17.2)	1 (2.0)	0.010
*seh*	1 (0.9)	1 (1.7)	0	1.000
*sei*	13 (12.1)	11 (19.0)	2 (4.1)	0.019
*sej*	3 (2.8)	3 (5.2)	0	0.304
*sasX*	0	0	0	–

*lukS/F-PV, gene encoding Panton-Valentine leukocidin*.

*tst, gene encoding toxic shock syndrome toxin 1*.

*eta and etb, gene encoding exfoliative toxin A and B*.

*sea-see and seg-sej, gene encoding staphylococcal enterotoxins SEA-SEE and SEG-SEJ*.

*sasX, gene encoding mobile genetic element*.

### Molecular typing

Thirty eight isolates belonged to SCC*mec* typeIV and 8 belonged to SCC*mec* typeIV. Three MRSA isolates could not be SCC*mec* typed. Forty *Spa* types were identified. t172 (18/107, 16.8%) was the most common, followed by t189 (13/107, 12.1%), t091, and t437 (10/107, 9.3%). t172 (17/49, 34.7%) and t189 (13/58, 22.4%) were the most common *spa* type in MRSA and MSSA respectively.

**Figure 1:**
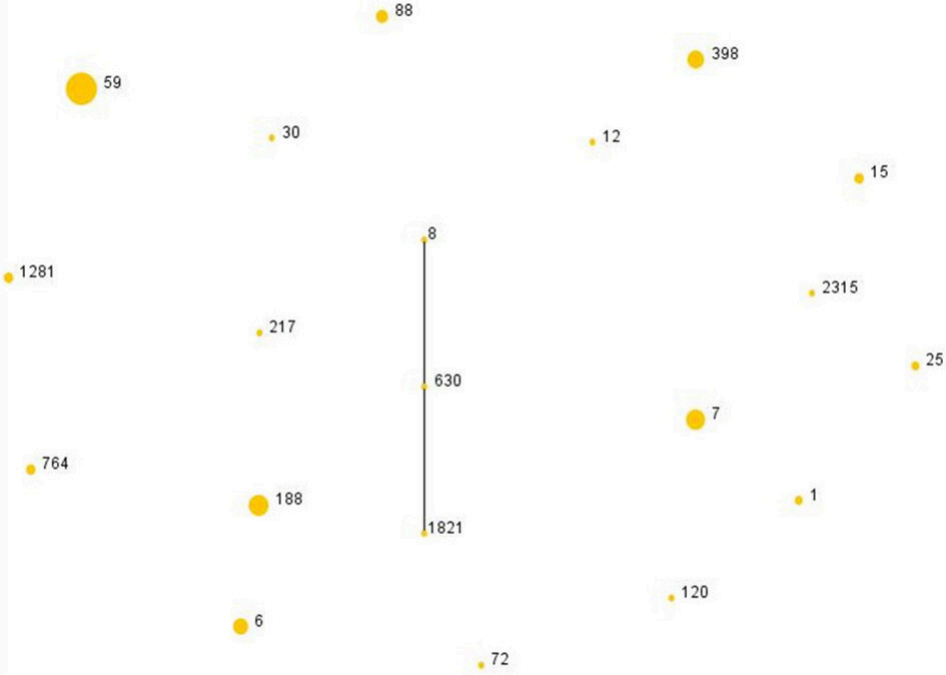
**The diagram generated by eBURST with the default group definition based on the MLST data of this study, illustrating the relations of 107 ***S. aureus*** isolates**. Each number implies an MLST ST and the dot area indicates the prevalence of the ST in the MLST data of this study.

Among all *S. aureus* isolates, 20 sequence types (STs) were identified by MLST. The most common ST was ST59 (35/107, 32.7%), followed by ST188 (14/107, 13.1%), ST7 (13/107, 12.1%), and ST398 (10/107, 9.3%). Eighteen clonal complexes (CCs) were identified, classified into one group (CC8) and 17 singletons by eBURST. CC59 (35/107, 32.7%) was the most common clone, followed by CC188 (14/107, 13.1%), CC7 (13/107, 12.1%) and CC398 (10/107, 9.3%) (Table 3 and Figure 1). ST59-SCCmec IV-t172 (17/49, 34.7%) was the most common clone in MRSA, followed by ST59-SCC*mec*IV-t437 (9/49, 18.4%). In ST59-SCCmec IV-t172 clone erythromycin, clindamycin and fosfomycin-trometamol had same resistance rate, which was up to 76.4%. Moreover, ST59-SCCmec IV-t437 clone showed high resistance to erythromycin, clindamycin and kanamycin as well, which was 88.9, 88.9, and 77.8% respectively. ST188-t189 (13/58, 22.4%) was the most common clone in MSSA, followed by ST7-t091 (10/58, 17.2%). Resistance rates of Penicillin in ST188-t189 and ST7-t091 were 92.3 and 90% respectively. Additionally, ST59 was the most frequent ST in *lukS/F-PV-*positive isolates, including 2 SCC*mec* typeV and 1 SCC*mec* type IV isolates. Other STs found in *lukS/F-PV-*positive isolates included ST217 (1MSSA), ST398 (1MSSA) and ST6 (1MSSA).

**Table 3:** **Molecular characteristics and antibiotic resistance profiles of ***S. aureus*** isolated from children with pneumonia**.

**CCs**	**ST (*n*)**	**MRSA (*n*)**	**MSSA (*n*)**	***agr* group (*n*)**	**spa type (*n*)**	**Virulence genes (*n*)**	**Antibiotic resistance profile (*n*)**
1	ST1(2)	IV(1)	1	III(2)	t114(1), t127(1)	*tst*(1), *sea*(1), *seh*(1)	P(2), FOT(2), CX(1), E(1), CL(1)
5	ST764(3)	NT(1)	2	II(3)	t002(3)	*tst*(1), *sea*(1), *seb*(1), *sec*(2), *sed*(2), *seg*(2), *sei*(3), *sej*(2)	P(3), CN(2), K(2), E(3), FOT(2), CC(2), CX(1), TOB(1), TE(1), MH(1), CIP(1)
6	ST6(8)	IV(4)	4	I(7), II(1)	t15317(1), t304(4), t4793(1), t701(2)	*sea*(4), eta(1), lukS/F-PV(1)	P(6), CX(4), FOT(5), E(5), CC(1)
7	ST7(13)	0	13	I(12), III(1)	t091(10), t605(2), t796(1)	eta(1), sea(1)	P(12), TOB(7), FOT(9), TE(5), E(3), K(2), CN(1), CC(1)
8	ST8(1), ST630(1), ST1821(1)	V(1)	2	I(3)	t9101(1), t4549(1), t3930(1)	*sed*(1), *sej*(1)	P(3), FOT(1), E(2), CX(1)
12	ST12(1)	0	1	II(1)	t15923(1)	*none*	P(1), FOT(1), E(1)
15	ST15(3)	0	3	II(2), III(1)	t085(1), t491(1), t774(1)	*none*	P(2), FOT(2), E(1)
20	ST1281(3)	0	3	I(3)	t164(2), t731(1)	*sec*(1), *seg*(2), *sei*(3)	P(2)FOT(2)
22	ST217(1)	0	1	I(1)	t309(1)	*lukS/F-PV*(1), *seg*(1), *sei*(1)	P(1), FOT(1)
25	ST25(2)	0	2	I(2)	t078(2)	*sec*(1), *seg*(2), *sei*(2)	P(2), E(2), TE(1)
30	ST30(1)	0	1	I(1)	t338(1)	*tst*(1), *sea*(1), *seg*(1), *sei*(1)	P(1), FOT(1), E(1)
59	ST59(35)	IV(31), V(2)	2	I(34), II(1)	t163(1), t172(18), t3523(1), t3736(1) t437(10), t441(3), t7281(1)	*sea*(20), *seb*(33), lukS/F-PV(3),	P(35), CX(33), FOT(18), E(28), CC(27), K(12), CL(2), TE(7), TOB(1)
72	ST72(1)	0	1	I(1)	t148(1)	*tst*(1), *seg*(1), *sei*(1)	None
88	ST88(5)	IV(2), NT(2)	1	III(5)	t1376(1), t15319(1), t2310(3)	*none*	P(5), FOT(4), CIP(1), CX(4), E(3), K(1), TOB(1)
120	ST120(1)	V(1)	0	IV(1)	t14775(1)	*eta*(1), *seg*(1), *sei*(1)	P(1), CX(1), FOT(1), E(1), CC(1)
188	ST188(14)	0	14	I(14)	t15294(1), t189(13)	*seb*(1), seb(7)	P(13), FOT(5), E(1)
398	ST398(10)	V(4)	6	I(10)	t034(7), t1456(1), t571(2)	*lukS/F-PV*(1)	P(9), FOT(10), CX(4), E(3)
2315	ST2315(1)	0	1	II(1)	t11687(1)	*seg*(1), *sei*(1)	P(1), FOT(1), CIP(1)

*CCs, clonal complexes; ST, sequence type by multi-locus sequence typing; SCCmec, Staphylococcal cassette chromosome mec; agr, accessory gene regulator; spa, Staphylococcusprotein A gene; NT, not-typeable; none, no virulence gene detected*.

*P, penicillin(10 units); CX, cefoxitin (30 μg); CN, gentamicin(10 μg); K, Kanamycin(30 μg); TOB, tobramycin (10 μg); FOT, Fosfomycin-trometamol(200 μg); E, erythromycin (15 μg); TE, tetracycline(30 μg); MH, minocycline(30 μg); CIP, ciprofloxacin(5 μg); CC, clindamycin (2 μg); CL, chloramphenicol(30 μg); None, sensitive to all tested drugs*.

Grouping of *agr* allele showed that *agr I* to *IV* was discovered in 87, 10, 9 and 1 isolates respectively. *agrI* (87/107, 81.3%) was the most common *agr* group, followed by *agrIII* (10/107, 9.3%), *agrII* (9/107, 8.4%), and *agr IV* (1/107, 0.9%). No significant difference was observed in the prevalence of *agr* allele between MSSA and MRSA.

## Discussion

The morbidity of childhood pneumonia caused by *S. aureus* particularly CA-MRSA has been increasing over the past two decades (Gonzalez et al., 2005; David and Daum, 2010). Among the 107 pediatric *S. aureus* pneumonia patients enrolled in current study, 49 isolates were CA-MRSA according to the definition by J. A. Otter et al and the epidemiological data in Asian pacific area (Ho et al., 2012; Otter et al., 2013; Xiao et al., 2013; Zhang H. et al., 2015). Unlike similar study conducted in Shanghai in 2005, which showed ST239, ST910, and ST88 were the main endemic *S. aureus* clones causing infections (Zhang et al., 2009), we found that CC59 was the most common CC, and it was also the most common CC among MRSA isolates in Shanghai. In 2009, Chen et al found HA-MRSA stains were still the major pathogen in healthcare- associated bloodstream or secondary to thermal injury in audit in Shanghai, and two main MRSA clones (ST239 and ST5) were prevailing in patients (Chen et al., 2012, 2013). ST239 was also reported as an epidemic clone causing *S. aureus* surgical site infections in orthopedic patients in Shanghai in 2011 (Gu et al., 2015a). Nevertheless, ST239 was not detected in our study, and CC8 (ST8, ST630, ST1821) was discovered in only 3 isolates. It seemed that ST59 had replaced ST239 in terms of dissemination in Shanghai. STs which proved capable of developing high-level of resistance to fluoroquinolones without suffering significant fitness cost would supplant others. However, other factors would determine clonal shifts in facilities/wards where fluoroquinolones are inappropriate in use (Fuzi, 2016). The dominant MRSA sequence type replacements are worth further studies. A study performed in 2008 showed ST59-SCCmec IV-t437 was the predominant clone of CA-MRSA which caused community –onset childhood pneumonia in China (Geng et al., 2010); however, the present study suggested that ST59-SCCmec IV-t172 had replaced ST59-SCCmec IV-t437 as the most common clone in CA-MRSA in Shanghai. Gu's study implied CC59 was the most common CC among adult patients with skin and soft tissue infections (SSTIs) in Shanghai in 2011 (Gu et al., 2015b). The study conducted among nursing home residents in Shanghai in 2014 showed CC1 was the most common clonal of *S. aureus* carriage (Zhang J. et al., 2015). This might indicate that CC59 is common in SSTIs and pneumonia, and will not be affected by population factors. CC398 was also recently reported as a livestock-associated clone in skin and soft-tissue infections (SSTIs) in China (Gu et al., 2015b) and the *spa* type t034 (CC398) is a typical livestock-associated *spa* type. In Europe, patients carrying this spa type are usually in contact with major animal reservoir (mostly pigs) carrying these MRSA (Köck et al., 2009). Livestock-associated *S. aureus* CC398 (ST398 with t034) was found in 7 isolates (4MRSA and 3 MSSA), and the SCC*mec* type of all CA-MRSA was V in this study. Clinical data on any possible children contact are not available in this study, but all children infected by ST398-t034 *S. aureus* were under 3 months. Transmission between animals and humans requires further research. ST188 (14/58, 24.1%) was the common genotype of MSSA in *S. aureus* pneumonia in Chinese children, which was in agreement with the results reported by Qiao et al. (2014).

The prevalence of enterotoxins among *S. aureus* (57.9%, 62/107) isolated from pediatric patients, is actually consistent with that from adult (53.8–65.9%) in the same region (Chen et al., 2013; Gu et al., 2015a,b). ETs positive strains in MRSA and MSSA were 35 (71.4%, 35/49) and 27 (46.6%, 27/58) respectively. The prevalence of ETs in MRSA was lower than the occurrence of MRSA from surgical site infections (80.1%, 29/36) and SSTI (92.9%, 13/14) patients and higher than bloodstream infection (59.7%, 37/62) patients as our previously studies in Shanghai (Chen et al., 2013; Gu et al., 2015a,b). MRSA tended to carry the *seb* and *sea* genes, while MSSA tended to carry *seg* and *sei* genes, which was similar with the findings of our study on *S. aureus* infecting surgical site in orthopedic patients (Gu et al., 2015a). Further studies are warranted to get better understanding of this epidemic phenomenon in Shanghai.

Panton-Valentine leukocidin (PVL) is a bicomponent toxin, causing the lysis of leucocytes by forming a pore in their membrane, and it is a main virulence factor of *S. aureus*, independent of methicillin resistance (Gillet et al., 2011), and responsible for severe necrotising pneumonia. In 2006, Geng et al. found 22 MRSA (22/50, 40%) stains that were PVL- positive from children with community-onset pneumonia in China. In the same year, Han et al isolated 9 MRSA strains harboring *lukS/F-PV* from 8 infants with pneumonia and 1 adult with prostatitis in Shanghai (Han et al., 2010). In Qiao's study the *pvl* gene was detected in 27% of the isolates in children with invasive CA-SA infections from 2011 to 2013 in China (Qiao et al., 2014). In current study, a total of six *lukS/F-PV* -positive isolates were identified, and it was lower than the occurrences of *lukS/F-PV*-positive *S. aureus* previously reported (Geng et al., 2010; Han et al., 2010; Qiao et al., 2014). Three of *lukS/F-PV* -positive isolates in CA-MRSA were ST59 and the remaining three were ST6, ST217, and ST398 MSSA respectively.

When CA-MRSA infections is suspected the initial empiric antibiotic therapy including vancomycin or Clindamycin will be applied according to the American Academy of Pediatrics' Committee on Infectious Diseases' recommendations in 2009 (American Academy of Pediatrics Committee on Infectious Diseases: Staphylococcal Infections et al., 2009). Current study showed linezolid and vancomycin were more susceptible. The results of antimicrobial susceptibility test suggested that linezolid and vancomycin were appropriate antibiotics for treating *S. aureus* including CA-MRSA childhood pneumonia. CA-MRSA strains are resistance to β-lactams and cephalosporins and mostly susceptible to several non-β-lactam antibiotics (Shilo and Quach, 2011; Otter and French, 2012). Surprisingly MRSA in our study revealed low susceptibility to erythromycin (18.4%) and clindamycin (40.8%), some of which are inducible resistance. So Clindamycin may not be the most suitable empirical treatment of CA-MRSA childhood pneumonia in Shanghai. All Seven isolates susceptible to penicillin were β-lactamase producers, precluding penicillin in their treatment. Fluoroquinolones are unsuitable drug for children with pneumonia in the pediatric wards. This may be an explanation of the relatively low rate of resistance to ciprofloxacin in MRSA isolates. What is noteworthy is that all ST59-SCCmec IV-t172 were ciprofloxacin-intermediate. The microbiology laboratory study is warranted for development of an effective antimicrobial regimen in this area.

## Author contributions

Conceived and designed the experiments: LH and PH. Performed the experiments: ZS and FG. Analyzed the data: YN. Contributed reagents/materials/analysis tools: LH, PH, and XG. Wrote the paper: ZS, FG, and LH.

## Funding

This study was supported by National Natural Science Foundation of China (grant numbers 81472010 and 81471908). This study was also supported by the Shanghai 3-Year Plan of the Key Subjects Construction in Public Health-Infectious Diseases and Pathogenic Microorganism (grant number 15GWZK0102).

### Conflict of interest statement

The authors declare that the research was conducted in the absence of any commercial or financial relationships that could be construed as a potential conflict of interest.
